# A multi-level annotated sensor dataset of gait freezing manifestations and severity in Parkinson’s disease

**DOI:** 10.1038/s41597-026-06645-1

**Published:** 2026-01-26

**Authors:** Luigi Borzì, Florenc Demrozi, Ruggero Angelo Bacchin, Cristian Turetta, Michele Tebaldi, Luis Sigcha, Samaneh Zolfaghari, Domiziana Rinaldi, Giuliana Fazzina, Giulio Balestro, Alessandro Picelli, Graziano Pravadelli, Gabriella Olmo, Stefano Tamburin, Leonardo Lopiano, Carlo Alberto Artusi

**Affiliations:** 1 Department of Control and Computer Engineering, Politecnico di Torino, Turin, Italy; 2 Department of Electrical Engineering and Computer Science, University of Stavanger, Stavanger, Norway; 3 Department of Neurosciences, Biomedicine and Movement Sciences, University of Verona, Verona, Italy; 4 Neurology Unit, Santa Chiara Hospital, Trento, Italy; 5 Department of Engineering for Innovation Medicine, University of Verona, Verona, Italy; 6 Department of Computer Science, University of Verona, Verona, Italy; 7 ALGORITMI Research Center, School of Engineering, University of Minho, Guimarães, Portugal; 8 School of Innovation Design and Engineering, Mälardalen University, Västerås, Sweden; 9 Department of Neuroscience, Mental Health and Sensory Organs, Sapienza University of Rome, Rome, Italy; 10 Sant’Andrea University Hospital, Rome, Italy; 11 Department of Neuroscience, University of Turin, Turin, Italy; 12 Neurology 2 Unit, A.O.U. Città Della Salute e Della Scienza Di Torino, Turin, Italy

**Keywords:** Biomedical engineering, Parkinson's disease, Parkinson's disease, Computer science

## Abstract

We present FoG-STAR, a dataset collected using wearable sensors, designed to support the development and evaluation of algorithms for detecting and characterizing freezing of gait (FoG) in people with Parkinson’s disease (PD). The dataset includes recordings from 22 participants who performed a series of standardized motor tasks while wearing four inertial measurement units (IMUs) on the ankles, wrist, and lower back. Each IMU recorded tri-axial accelerometer and gyroscope data. Participants completed seven structured tasks, including walking with/without cognitive/motor dual-tasks, 360-degree turning, and the timed-up-and-go test, which comprises six types of activities (sitting, standing, sit-to-stand, stand-to-sit, walking, and turning). The dataset features detailed annotations from two expert clinical raters, who marked the onset and offset of 101 FoG episodes, and labelled specific FoG manifestations. In addition, the duration of each activity and task segment was annotated. This multi-level annotation framework allows for studying FoG in the context of dynamic motor behavior and provides a valuable resource for the development of machine learning models aimed at FoG detection, severity assessment, and activity recognition in PD.

## Background & Summary

Parkinson’s disease (PD) is a progressive neurodegenerative disorder that affects approximately 2% of people over the age of 60^1,2^. It is characterized by core motor symptoms such as resting tremor, rigidity, and bradykinesia, along with a wide range of non-motor impairments including cognitive decline, sleep disturbances, and autonomic dysfunction^2,3^. While initial treatment strategies—most notably the administration of Levodopa—can provide symptom relief, the progression of the disease typically leads to motor complications such as dyskinesias and fluctuations in motor performance, which significantly reduce quality of life^3,4^. One of the most disabling manifestations of motor impairment is freezing of gait (FoG), a transient and unpredictable failure to initiate or maintain effective walking, often described as a sensation of the feet being “glued to the floor”^5^. FoG affects nearly 50% of people with PD and up to 80% in advanced stages^6^. It is a major contributor to falls, reduced mobility, and loss of independence^7,8^. The underlying cause of FoG is not fully understood, but is generally considered to result from impaired coordination between motor and cognitive processes, particularly during high-demand situations such as turning, initiation of gait, or navigating narrow spaces^5^. Rather than viewing FoG solely through the lens of neurophysiology, recent research increasingly considers it a complex behavioral event that can be quantitatively characterized using sensor data. The episodic, irregular, and highly individual-specific manifestation of FoG makes it difficult to capture and quantify through traditional clinical observation. These challenges have motivated the use of wearable sensing systems and computational modeling to support objective detection and prediction^9^. Wearable devices—such as inertial measurement units (IMUs), pressure sensors, and surface electromyography (sEMG)—enable the collection of detailed kinematic and physiological data in both controlled and naturalistic environments^10,11^. These sensor-rich datasets have proven useful for training machine learning (ML) and deep learning (DL) models that aim to classify FoG events, detect pre-freezing gait patterns, and even predict FoG onset in real time^12^. However, FoG is not a straightforward target for ML. Its temporal variability, sparse event frequency, and inter-patient heterogeneity pose major challenges to model generalizability^13,14^. DL approaches, particularly those based on recurrent or convolutional neural networks (CNNs), have shown promise in capturing the sequential and spatial structure of gait data^15–17^. Still, their effectiveness is highly dependent on the quality and quantity of labeled data. Recent studies have begun to address FoG severity and manifestations using wearable-sensor data^18,19^, underscoring the need for datasets that include similarly fine-grained annotations. Nevertheless, publicly available datasets providing detailed severity and manifestation-level annotations remain limited, underscoring the need for resources that support both FoG-specific analyses and broader examinations of motor behavior in PD. Therefore, efforts to build robust, reproducible systems focus on collecting large-scale, multimodal datasets and designing domain-aware architectures that incorporate temporal dynamics and contextual features^20,21^. Ultimately, improving the detection and forecasting of FoG through data-driven methods may enable more personalized intervention strategies and real-time feedback systems, contributing to improved mobility, safety, and overall quality of life for people with PD^16,22^.

Some open-access datasets have been established to support the development and evaluation of FoG detection methods in people with PD using wearable devices. Table 1 provides an overview of existing datasets, summarizing key aspects such as the number of subjects, type and placement of sensors, tasks performed, marked clinical events, and availability of clinical information. Together, these datasets provide a robust foundation for advancing objective FoG analysis, comparing sensor configurations, and exploring multimodal approaches that integrate motor, cognitive, and affective dimensions of FoG episodes. Their diversity also supports algorithm generalization and practical deployment in both clinical and free-living environments. Indeed, prior studies have conducted multi-site evaluations of ML and DL algorithms, revealing the need for models that generalize beyond their training cohort and experimental settings^14,23^.

**Table 1:** Overview of publicly available datasets on FoG.

Dataset (year)	Subjects	#Devices (type)	Sensor location	#Tasks (activities)	Marked events	Clinical info
Daphnet^24^ (2010)	10	3 (acc)	Shank, thigh, lower back	3 (Walking, turning, stopping, ADL)	FoG	Gender, age, disease duration, H&Y
Multimodal^25^ (2021)	12	4 (acc, EEG, EMG, SC)	Waist, l-shank, r-shank, wrist	2 (Standing up, walking through obstacles or narrow spaces, turning, sitting)	FoG	Gender, Age, Disease duration, UPDRS, FOG-Q, MMSE, MOCA
IMU^26^ (2022)	35	1 (acc, gyro)	Ankle	1 (360 Turn left and right)	FoG	Gender, age, disease duration, H&Y, UPDRS, NFOG-Q, MMSE, MOCA, FAB, HADS
Oday^27^ (2022)	7	11 (acc,gyro)	Head, chest, back, wrists, thighs, ankles, feet	1 (Walking trial, turning, obstacles)	FoG	Gender, age, disease duration
tDCS^30^ (2023)	62	1 (acc)	lower back	7 (TUG, Walking, turning, passing trough a door, dual tasks)	FoG, FoG trigger (walking, start hesitation, turning)	Gender, Age, Disease duration, UPDRS, NFOG-Q, Mini-BEST
WearGait^29^ (2024)	61	13 (acc, gyro), pressure insoles	Head, chest, back, wrists, thighs, shins, ankles, feet	Structured and semi-structured gait and balance tasks	FoG, Clinical events (attempt to rise, hesitation)	Age, sex, MDS-UPDRS, H&Y, DBS status, medication state
Delgado^28^ (2025)	21	1 (acc, gyro)	Ankle	Semi-structured daily activities (walking, turning, standing, sitting)	FoG	Age, sex, disease duration, MDS-UPDRS, Mini-BEST, MOCA
FoG-STAR (2025)	22	4 (acc, gyro)	Lower back, l-ankle, r-ankle, wrist	7 (Walk, turn, sitting, standing, stand-up, sit-down)	FoG, FoG manifestation, Activity (walk, turn, sit, stand, sit-to-stand, stand-to-sit)	Age, gender, disease duration, H&Y stage, UPDRS-III FOG-Q, MOCA, FES-I, PDQ-8

acc: accelerometer; gyro: gyroscope; ADL: activities of daily living; EEG: electroencephalography; EMG: eletromyography; SC: skin conductance; TUG: timed-up-and-go; FoG: freezing of gait; H&Y: Hoehn and Yahr; UPDRS: Unified Parkinson’s Disease Rating Scale; NFOG-Q: New FoG Questionnaire; DBS: Deep Brain Stimulation; MMSE: Mini-Mental State Examination; MOCA: Montreal Cognitive Assessment; FAB: Frontal Assessment Battery; HADS: Hospital Anxiety and Depression Scale; FES-I: Falls Efficacy Scale-International; PDQ-8: Parkinson’s Disease Questionnaire.

All of these public FoG datasets share strengths and limitations. Daphnet^24^ has been the most widely used FoG dataset in the literature, supporting the development of several detection methods over the past decades^9^. However, it comprises a small cohort of 10 subjects and only acceleration sensors. Multimodal^25^ offers multimodal data (skin conductance, electroencephalography, electroencephalogram, inertial signals) to improve model performance. However, data were collected from a small sample of 12 subjects. IMU^26^ includes a larger cohort of 35 subjects. However, data were only recorded from the 360-degree turning task and using a single sensor on the ankle. Oday^27^ includes 13 sensors on the body and different activity tasks. However, it only includes 7 subjects. Delgado^28^ includes a larger sample and a wider range of activities, performed in less supervised settings. However, the data were recorded from a single sensor on the ankle. All of these datasets share a significant limitation, which is that they only have labels related to FoG. No information is available on events or activities outside of FoG events. Recent datasets have taken a step toward more contextual FoG information. WearGait^29^ includes 61 subjects with PD and 13 sensors placed on different parts of the body. In addition, some clinical events are marked outside the FoG, including attempts to stand up and start hesitations. tDCS^30^ includes data recorded from a single accelerometer on the back. However, the dataset includes a large cohort of 62 subjects and several motor tasks. Furthermore, it represents the first dataset to provide accurate labeling of FoG triggering situations (start hesitation, turning FoG, straight-line FoG). Despite this significant advantage, activities or events outside FoG events are not labeled.

FoG-STAR: Freezing of Gait Severity, Tasks, Activities, and Ratings dataset provides several key advancements over existing databases. First, it includes synchronized measurements from multiple IMUs (accelerometer and gyroscope) placed on representative body segments, namely the lower limbs (ankles), the center of mass (lower back), and the upper extremities (wrist), thereby enabling a more comprehensive characterization of whole-body movement during FoG episodes. Second, the dataset comprises recordings from 22 individuals with PD, accompanied by detailed demographic and clinical information to support stratified analyses. Most importantly, the dataset offers rich and fine-grained annotations, including FoG occurrence and manifestation, structured motor tasks (e.g., standing, 360-degree turning, Timed Up and Go), and everyday activities (stand-up, sit-down, stand, sit, walk, turn), both during and outside FoG events. This level of contextual and continuous labeling provides a broader view of patient mobility and addresses a major limitation of prior datasets, which typically only annotate FoG events. Together, these features make this dataset a valuable resource for developing and benchmarking robust, context-aware FoG detection and prediction algorithms.

The FoG-STAR dataset has already been used in preliminary research^31^ to investigate the impact of sensor type, placement, and activity protocols on ML-based FoG detection. This study systematically assessed accelerometer and gyroscope data across different body locations (wrist, ankles, and lower back) and task conditions, demonstrating the dataset’s value for benchmarking FoG detection methods. These initial results underscored the importance of multi-sensor configurations and diverse activity protocols, while establishing FoG-STAR as a robust resource for evaluating wearable sensing and artificial intelligence (AI) approaches in PD. FoG-STAR complements existing resources by providing a balanced contribution to the FoG dataset landscape. The main contributions include: (i) a diverse set of daily-life-related tasks, enabling both FoG detection and broader activity recognition; (ii) fine-grained annotations that capture FoG severity/manifestation and contextual activity information; and (iii) extensive clinical assessments, including UPDRS-III, FoG-Q, MoCA, and quality-of-life questionnaires. Together, these features make FoG-STAR a well-curated benchmark for advancing ML methodologies and supporting clinically oriented studies on the multifaceted nature of FoG in PD.

The remainder of the paper is organized as follows. Section Methods describes the enrollment procedures, participants’ demographic and clinical information, body sensor network, data acquisition protocol, and data processing steps. Section Data Records presents the dataset organisation and composition, and describe the dataset characteristics. Finally, Section Technical Validation details the technical validation of the dataset.

## Methods

### Experimental design

#### Participants

Individuals with idiopathic PD, diagnosed according to the international Movement Disorder Society (MDS) clinical diagnostic criteria, were recruited from two Italian university movement disorders clinics: the Department of Neurosciences and Mental Health, University Hospital Trust of Torino (Turin), and the Department of Neurosciences, Biomedicine and Movement Sciences, University of Verona (Verona). The inclusion criteria are summarized in Table 2, while the exclusion criteria included the presence of dementia or significant musculoskeletal, cardiovascular, psychiatric, or other neurological conditions affecting gait.

**Table 2:** Inclusion criteria for participant recruitment.

Criterion	Description
Diagnosis	Idiopathic PD according to the MDS clinical diagnostic criteria^42^
Disease stage	H&Y score between 2 and 4
FoG	Daily FoG episodes in the past month, with NFoG-Q score of 1 on Question 1 and ≥2 on Question 2^43^
Treatment stability	Stable PD medication for more than one month prior to enrollment
Walking ability	Able to walk continuously and independently for at least 15 minutes (use of cane or rollator permitted)

MDS: Movement Disorder Society; H&Y: Hoehn and Yahr stage; NFoG-Q: New FoG Questionnaire.

The study enrolled 22 people with PD, whose demographic and clinical characteristics are summarized in Table 3. To minimize medication effects, participants were instructed not to take their last dose of Levodopa the day before testing, to ensure evaluation after a 12-hour drug washout. Clinical evaluations were performed in the morning, immediately followed by sensor-based data collection. Written informed consent was obtained from all participants, including permission for the use and sharing of study data in anonymised form. The study was approved by the Ethics Committee for Clinical Trials of the Provinces of Verona and Rovigo (approval no. 3670CESC) and by Città della Salute e della Scienza di Torino (approval no. 0086153), Italy, which confirmed the possibility to use and publish the data in their current anonymised form. The study was carried out following the ethical principles outlined in the Declaration of Helsinki.

**Table 3:** Variables for demographic and clinical-related data.

Age (years)	Gender	Disease duration (years)	H&Y	UPDRS-III	FOG-Q	MOCA	FES-I	PDQ-8
70.9 ± 9.3	13M, 9F	10.1 ± 6.8	2.7 ± 0.8	38.9 ± 14.6	18.3 ± 5.4	21.3 ± 3.9	36.9 ± 26.9	14.4 ± 6.9

H&Y: Hoehn and Yahr stage; UPDRS: unified Parkinson’s disease rating scale; FOG-Q: FoG questionnaire; MOCA: Montral cognitive assessment; FES-I: Fall efficacy scale international; PDQ-8: Parkinson’s disease questionnaire.

#### Wireless Body Area Network

Participants were instrumented with a wireless body area network (WBAN)^32^ consisting of four IMUs (Nordic Thingy:52, Nordic Semiconductor). Each device is compact (6 × 6 cm, 47 g) and integrates a 9-axis motion-sensing module (3-axis accelerometer, 3-axis gyroscope, and compass) along with Bluetooth Low Energy (BLE) communication and an internal power supply. While the hardware includes additional environmental sensors (microphone, humidity, temperature, pressure, gas, color, and light), only the accelerometer and gyroscope signals were used in this study. Although the Nordic Thingy:52 platform is primarily a prototyping device, the embedded inertial sensor is the MPU-9250 (TDK InvenSense), a widely adopted 9-axis IMU used in numerous research-grade and commercial motion-analysis systems. The MPU-9250 has undergone extensive manufacturer validation and has been employed in several clinical and biomechanical studies, demonstrating stable performance with low drift and consistent signal quality. In our dataset, we did not observe abnormal drift or excessive noise beyond the expected behavior. Technical specifications are summarized in Table 4.

**Table 4:** Technical specifications of the IMUs used in the WBAN.

Feature	Specification
Device model	Nordic Thingy:52
Dimensions	6 × 6 cm
Weight	47 g
Motion sensors	MPU-9250 (TDK InvenSense): 3-axis accelerometer, 3-axis gyroscope, compass
Other sensors (not used)	Microphone, humidity, temperature, pressure, gas, color, light
Sampling rate	60 Hz (accelerometer and gyroscope)
Accelerometer range	±2*g* to ±16*g* (sensitivity: 4800 LSB/*g*)
Gyroscope range	±250^°^/*s* to ±2000^°^/*s*
Data transmission	Bluetooth Low Energy
Data collection	Smartphone gateway (Realme Pro 7)
Video recording	30 fps, synchronized with IMU data for annotation

**Fig. 1:**
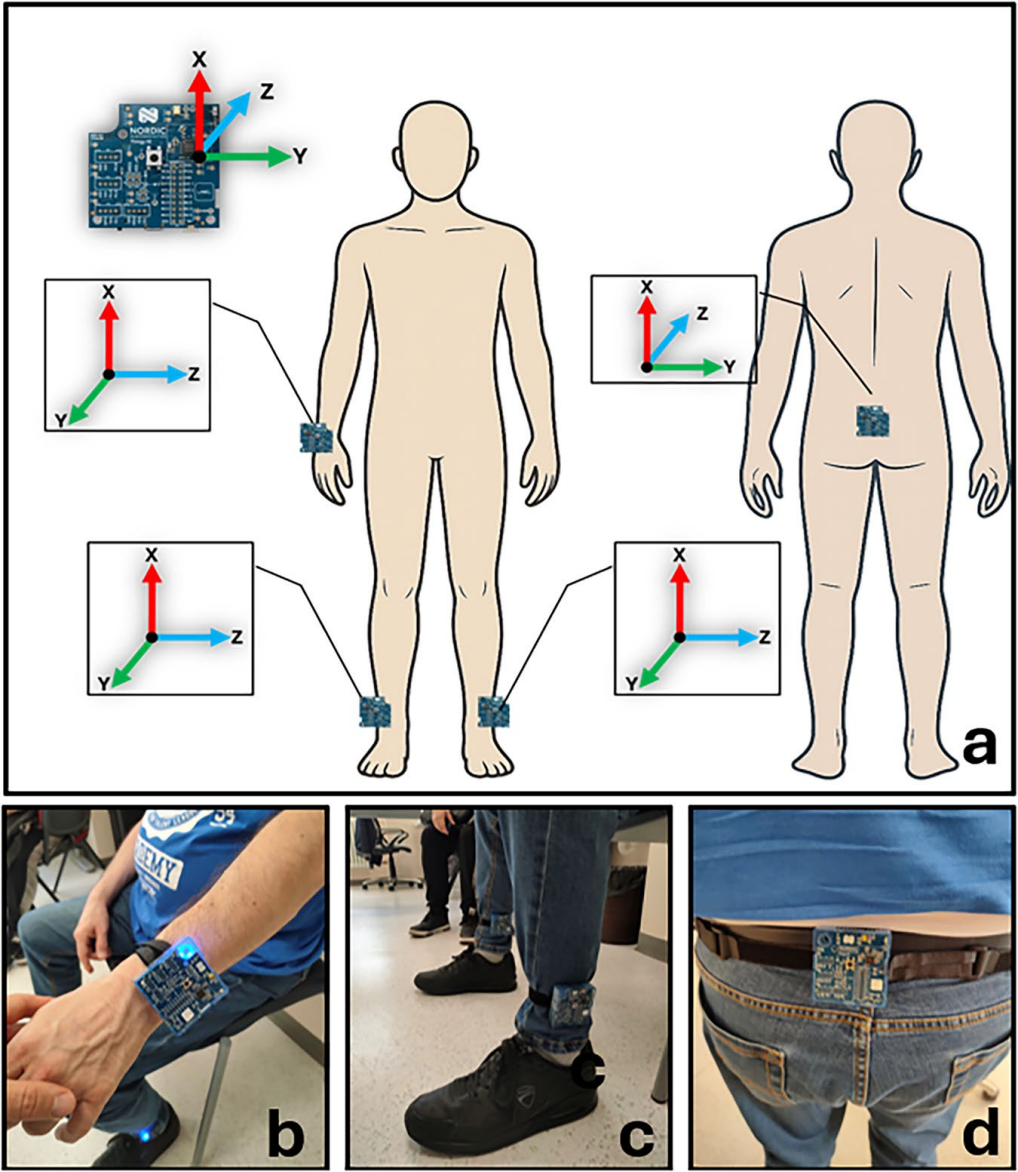
Sensor placement configuration for the dataset acquisition. (**a**) Schematic rendering with orientation of the local coordinate systems for each sensor. (**b**) Real-world sensor attachment examples: wrist, (**c**) ankles, and (**d**) lower back.

IMU placement on the body is illustrated in Fig. 1a: one unit was positioned on the wrist of the most affected side (1b), two on the lower legs just above the ankles (1c), and one on the lower back (L3-L5 level) (1d). This configuration enabled the capture of detailed motion patterns from both lower and upper body segments, synchronized with video recordings to support precise annotation. Participants expressed high satisfaction with the wearable technology employed for data acquisition, as reflected by a Quebec User Evaluation of Satisfaction with Assistive Technology (QUEST)^33^ device score of 38.2 ± 1.9 (range: 34–40), where 40 represents the maximum satisfaction rating. Each of eight device-related aspects—comfort, weight, durability, adjustability, ease of use, dimensions, effectiveness, and safety—was rated on a 0-5 scale.

### Data collection

Participants performed a set of standardized motor tasks, with self-paced rest intervals between trials. Clear instructions were provided before each task, and participants were free to withdraw from the study at any time. The experimental protocol included seven tasks covering daily activities, gait with dual-task conditions, and turning maneuvers, as detailed in Table 5. All participants took part in the experiments in the “Off” condition, after a 12-hour washout period from the drug. This was done to increase the likelihood of observing FoG episodes.

**Table 5:** Tasks included in the experimental protocol.

Task ID	Activity
1	Timed up and go test
2	Keep a static upright position with eyes open for 1 minute
3	Walk back and forth for 10 meters
4	Walk back and forth for 10 meters with passage through a doorway
5	Walking back and forth for 10 meters carrying a glass full of water
6	Walking back and forth for 10 meters counting backward from 100 to 0 with steps of 7
7	360 degree turn

### Data processing

#### Video annotation

Two neurologists (CAA, RAB), both specialists in PD and movement disorders, independently annotated activities and FoG episodes by carefully reviewing synchronized video recordings, following a standardized assessment protocol. The definition of FoG (inability to produce effective steps) and its subtypes–start hesitation, turn hesitation, FoG during turning, hesitation in narrow spaces, and destination or open-space hesitation– and manifestations were adopted from prior studies^34,35^.

FoG onset was defined as the frame corresponding to the last effective step before freezing, and offset as the first effective step afterward. Each episode was annotated using three FoG categories–shuffling, trembling in place, and akinesia (Table 6)–which are conventionally described as distinct manifestations of FoG^35^. In the present study, we use these manifestations as an ordinal indicator of episode-level severity, with increasing degree of movement arrest observed from shuffling to trembling to complete akinesia. Because different manifestations may appear sequentially within a single episode, adjacent segments of the same episode can receive different severity labels.

**Table 6:** Manifestations of FoG and the corresponding episode-level severity score assigned.

Severity	Manifestation
1	Shuffling forward with small steps
2	Trembling in place with alternating rapid knee movements
3	Complete akinesia, no limb or trunk movement

To simplify annotation, video recordings were downsampled to 10 fps, ensuring a temporal resolution of 100 ms for both activity and FoG identification. Annotations were performed using the Python Video Annotator software^36^ and exported in CSV format for subsequent analysis.

The intra-class correlation coefficient (ICC) between the two raters was 0.80 for the number of FoG episodes and 0.88 for the percent time spent with FoG, which show good-to-strong inter-rater agreement^37,38^. Discrepancies between raters were resolved through consensus. A conflict was flagged when (i) one rater marked an episode not identified by the other, (ii) overlap between annotated episodes was below 80%, or (iii) onset times differed by more than 0.5 s. For episodes with high inter-rater agreement, the final FoG onset and offset were computed as the average of both annotations.

#### Sensor synchronization

**Fig. 2:**
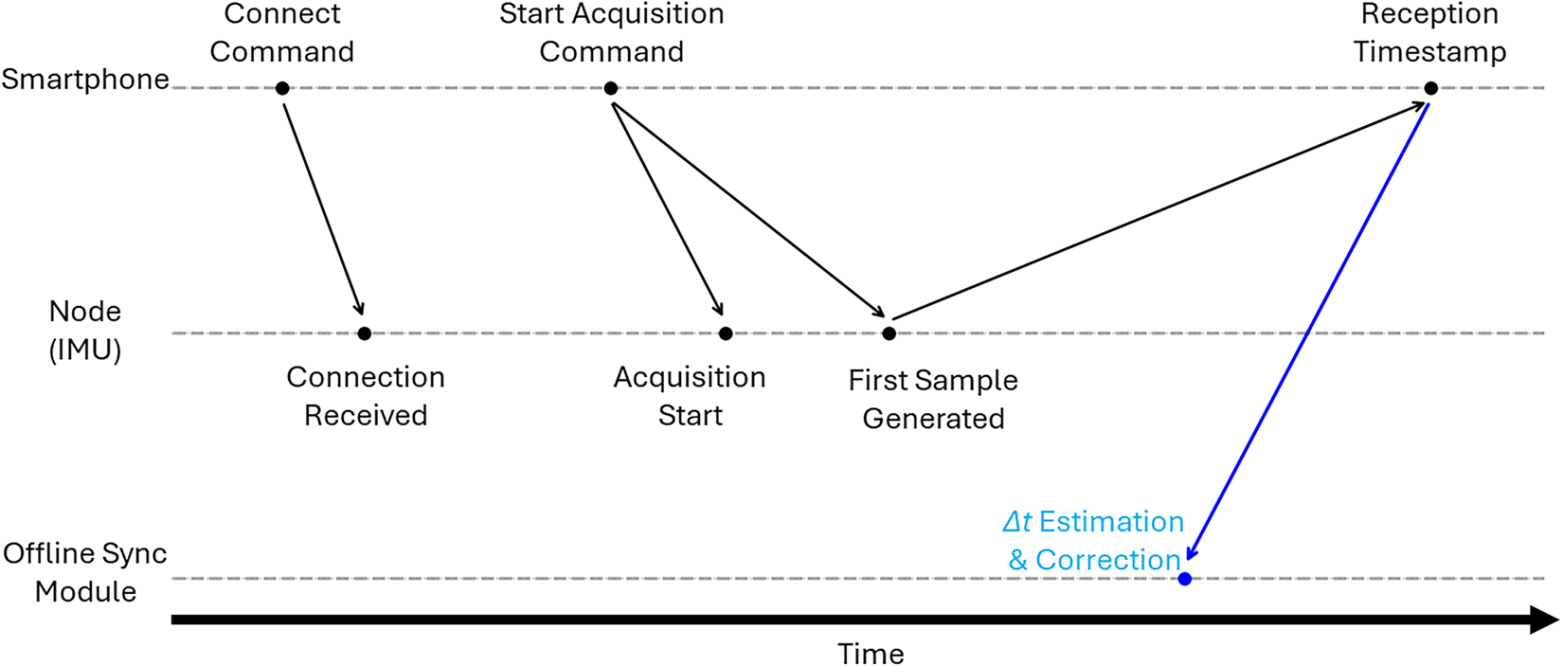
Overview of the synchronization workflow. Commands are issued by the smartphone, processed by the IMU nodes with intrinsic delays, and aligned offline by estimating a time offset (*Δt*) to reconstruct the actual sample generation time.

In the WBAN, the individual nodes did not communicate directly with each other and no native synchronization mechanism with the central aggregator (smartphone) was available. Therefore, synchronization of the IMUs was performed offline using an empirical procedure^32^. Specifically, we implemented a post-processing module that estimated, for each node, the delay between the smartphone’s transmission of the “connect” and “start acquisition” commands and the actual start of data collection at the node. Since all data samples were timestamped upon reception by the smartphone, we combined this information with the estimated command delays to back-calculate the approximate generation time of each sensor sample at the node. The correction was achieved by computing a synchronization offset (*Δt*), defined as the time difference between the node’s effective sensing start and the smartphone’s reception time. This *Δt* was then subtracted from the smartphone timestamps to realign samples across all nodes. The procedure relied on knowledge of the predefined sensor sampling frequency (60 Hz) and typical Bluetooth transmission latencies to refine the alignment of signals. Because BLE transmission latency can fluctuate slightly over time, we estimated the synchronization offset (*Δt*) once at the beginning of each recording session. The offset was computed from the delay between the smartphone’s command timestamps and the arrival of the first valid samples from each IMU. Although BLE latency is not perfectly constant, the observed variability remained within a few milliseconds, substantially smaller than the 16.7 ms sampling period of the sensors. For this reason, applying a continuously updated (dynamic) correction was unnecessary. The single-session offset provided stable alignment among sensors throughout the recordings, and all reconstructed timelines showed consistent inter-device synchronization. The synchronization workflow is illustrated in Fig. 2.

It is important to note that the offline synchronization module does not record a physical timestamp during data acquisition. Instead, it computes a post hoc correction offset (*Δt*) by combining the smartphone reception timestamps with the known sampling rate and the estimated BLE transmission delay. This offset is then subtracted from the reception timestamps to reconstruct the approximate sample-generation time on each IMU. Therefore, the timestamp shown in Fig. 2 reflects the moment at which the correction is computed offline, not the actual moment at which the sample was generated on the device.

Although we did not compute empirical synchronization error on a per-sample basis, the expected alignment accuracy can be derived from the characteristics of the BLE protocol and the MPU-9250 sampling hardware. BLE 4.x transmission introduces a typical jitter of approximately 1-3 ms depending on channel conditions and packet scheduling, while the MPU-9250 exhibits sub-millisecond timing jitter relative to the nominal 60 Hz sampling rate according to manufacturer specifications. Therefore, the expected inter-IMU synchronization uncertainty after applying the single-session *Δt* correction is on the order of 2-5 ms, which is significantly smaller than the 16.7 ms sampling interval. This level of variability is negligible for gait-cycle timing and FoG event characterization at the temporal granularity used in this study.

Finally, as defined in^32^, the IMU data and video recordings were automatically synchronized because both streams were captured by the same smartphone. The custom acquisition app simultaneously recorded video using the phone’s internal camera and received BLE packets from all IMUs, assigning reception timestamps using the phone’s system clock. This ensures that both video frames and sensor samples share a common time base. During the annotation process, video frame timestamps were directly aligned with the IMU data, enabling frame-accurate identification of FoG onset and offset without requiring external synchronization hardware.

## Data Records

The dataset is deposited and available at https://zenodo.org/records/17838806^39^. To ease the dataset exploration, visualization, and analysis, data were organized in two *CSV* files: *Sensor Data* and *Clinical Data*.

### Sensor data

The file *sensor_data.csv* includes the sensor readings from all experiments. The file has *m* = 329,027 rows/samples and *n* = 31 columns/variables. This file includes all the data needed to explore the dataset, visualize patterns, compare signals from different subjects or tasks, and train and evaluate ML/DL models. Variables include the *timestamp* (*F*_*s*_ = 60 Hz); 24 *sensor readings* variables, made up of 6 channels (3-dimensional acceleration and angular velocity data measured in g-force and degree/s, respectively) recorded by each of the 4 IMUs; the *activity* label indicating the activity class for each sample; the *FoG* label indicating the presence of FoG in each sample; and *FoG severity*, describing the severity of FoG in each sample. It is worth noting that activity and FoG labels are stored as independent binary/categorical variables and can overlap in time. Three additional columns report the ID of the *subject*, *session*, and *task* for each sample. A detailed description of the file is provided in Table 7. It should be noted that the activity and FoG labels are independent and may overlap. Each sample associated with a FoG event has a corresponding activity class, which provides context for the FoG manifestation.

**Table 7:** Variables for sensor-related data.

Column	Variable	Description
1	*Timestamp*	Float number corresponding to the estimated (as shown in Fig. 2) timestamp in milliseconds. The sampling rate is equal to 60 Hz.
2–25	*Sensor* *readings*	Float numbers representing inertial signals, stored in the following format: [*sensor position*]_[sensor type]_[direction], where the sensor position includes left (ankleL) and right ankle (ankleR), back, and wrist; sensor type includes accelerometer (acc) and gyroscope (gyro); and the direction comprises x,y, and z. The unit of measurement for the accelerometer and gyroscope readings is g-force and degree/s, respectively.
26	*activity*	Integer number that identify the specific activity performed. It includes 1: walking, 2: sit, 3: stand, 4: sit-to-stand, 5: stand-to-sit, 6: turn-right, and 7: turn-left.
27	*fog*	Binary value that identify the presence of FoG. It can be either 0 (non-FoG) or 1 (FoG).
28	*fog_severity*	Integer number indicating the severity of FoG, including 1: shuffling forward with small steps, 2: trembling in place with alternating rapid knee movements, and 3: complete akinesia without limbs or trunk.
29	*subjectID*	Integer number in the range from 1 to 22 that identify the specific subject. This number corresponds to that in the related demographic and clinical information.
30	*sessionID*	Integer number referring to the data acquisition session. In most cases, a single session was recorded for each subject. In some particular cases (e.g., issues in sensor synchronization), additional session (s) were registered.
31	*taskID*	Integer number corresponding to the specific motor task. It includes 1: timed up-and-go, 2: stand for 1 minute, 3: walk back and forth, 4: walk back and forth with passage through a doorway, 5: walk back and forth carrying a glass full of water, 6: walk back and forth counting backward, 7: 360 degree turn. Not all tasks are available for each subject.

### Clinical data

A second file, named *clinical_data.csv* contains the demographic and clinical information of each participant. The file is organized into *m*=22 rows, each representing a subject, and *n*=10 columns/variables containing different variables. The *subjectID* variable indicates the numerical identifier for each subject and matches the *subjectID* in the *sensor_data.csv* file, facilitating quick and easy data linkage. This file includes demographic (i.e., *age*, *gender*) and clinical information, including *disease duration* and progression (i.e., *h_y*), along with several overall scores derived from clinical examination and questionnaires administered to participants. Specifically, motor performance was assessed using the MDS-UPDRS part III (as *updrs_iii*); FoG frequency and severity through the *FoG-Q*; cognitive impairment through the *MOCA*; fear of falling assessed with *FES-I*; and quality of life measured by the *PDQ-8*. A detailed description of this file is provided in Table 8.

**Table 8:** Variables for demographic and clinical-related data.

Column	Variable	Description
1	*subjectID*	Integer number in the range from 1 to 22 that identify the specific subject. This number corresponds to that in the related sensor data.
2	*age*	Integer number referring the partipant’s age.
3	*gender*	Categorical value (M/F) corresponding to the participant’s gender.
4	*disease_duration*	Integer number reporting the number of years from the clinical diagnosis.
5	*h_y*	H&Y stage. Float number between 0 and 5, proportional to the disease progression.
6	*updrs_iii*	Integer number from 0 to 76, corresponding to the sum of all items from the MDS-UPDRS part III. Higher scores correspond to more severe motor impairment.
7	*fog_q*	Integer number from 0 to 24, calculated as the total score from the FOG-Q. Higher scores correspond to more severe FOG.
8	*moca*	Integer number from 0 to 30, calculated as the total MOCA score. Lower scores correspond to more severe cognitive decline.
9	*fes_i*	Integer number between 16 and 64, obtained as the total score from the FES-I. Higher scores correspond to more severe concern about falling.
10	*pdq_8*	Integer number between 0 and 32, calculated as the sum of all items from the PDQ-8. Higher scores signify poorer quality of life.

## Data Overview

**Fig. 3:**
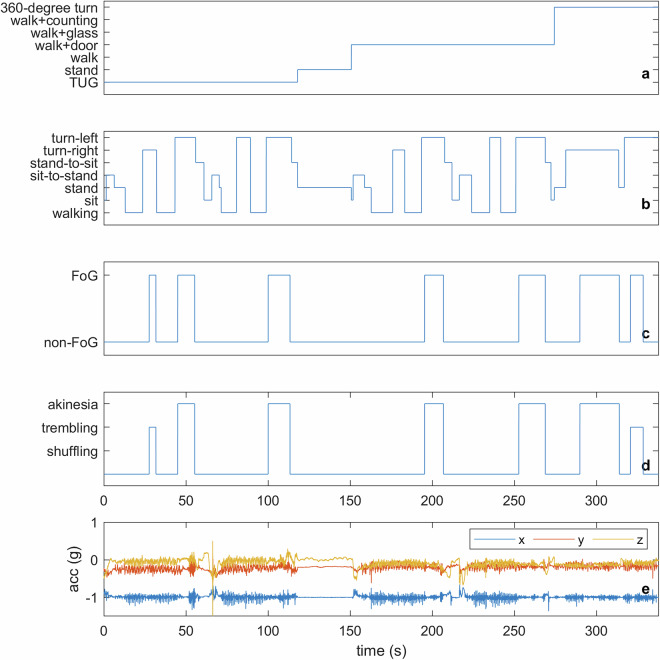
Representative synchronized view of the dataset. From top to bottom: task (**a**), activity (**b**), binary FoG label (**c**), FoG severity (1–3)/manifestation (shuffling, trembling, akinesia) (**d**), and raw acceleration from the lower-back IMU (**e**). This figure illustrates how annotations align with sensor signals and can be reproduced with the provided scripts.

The dataset offers a detailed representation of motor activities and FoG episodes in individuals with PD. To help users comprehend its structure, we provide the distribution of recording times and the severity of FoG events. Rather than presenting an analysis, the goal is to offer representative views for a better understanding of the data’s structure and scope. Figure 3 shows a representative trace with synchronized labels (task, activity, FoG, severity) overlaid on 3-axis lower back acceleration.

**Fig. 4:**
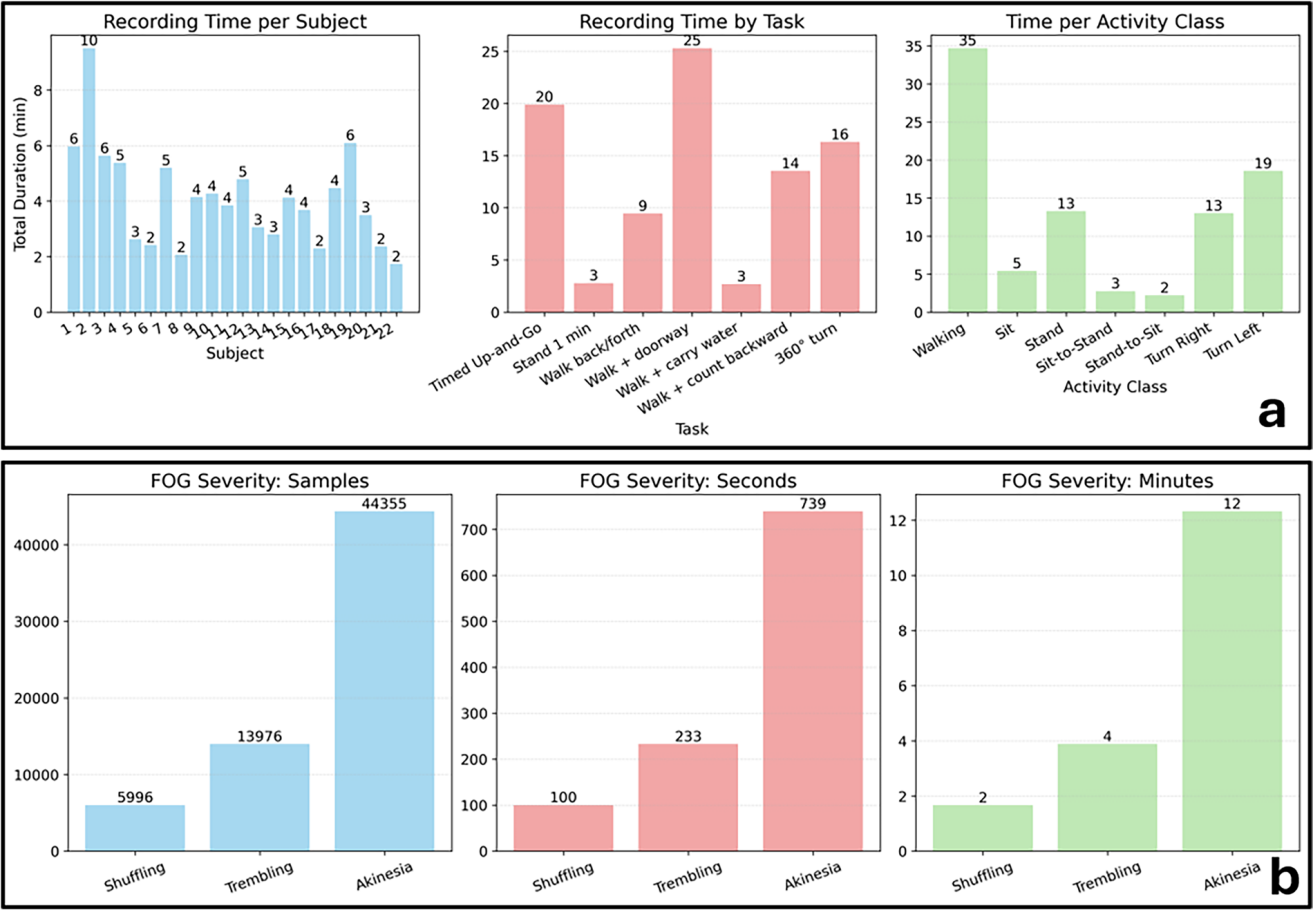
Dataset overview. (**a**) Recording time distributions across subjects (left), tasks (center), and annotated activity classes (right); each bar indicates the total duration of recordings, highlighting variability across participants and protocol tasks. (**b**) Distribution of FoG severity levels—shuffling, trembling, and akinesia—quantified in annotated samples (left), cumulative seconds (center), and minutes (right). Together, the panels illustrate the dataset composition in terms of both motor activity coverage and FoG manifestations.

Figure 4a illustrates the recording time distribution across three levels: (i) by subject, showing inter-individual variability in total duration (mean: 4.2 min, std: 1.8 min); (ii) by task, indicating that most of the time was spent in walking-related tasks and the “360^°^ turn”; and (iii) by activity class, highlighting the prevalence of walking, turning, and standing compared to postural transitions such as sit-to-stand. This characterization underscores the dataset’s balanced representation of clinically relevant tasks while reflecting natural variability across subjects.

Figure 4b focuses on FoG severity annotations. Episodes are categorized into three manifestations—shuffling, trembling, and akinesia—and are presented as total annotated samples, cumulative duration in seconds, and equivalent duration in minutes. The distributions show that akinesia constitutes the largest portion of FoG time, whereas shuffling and trembling represent shorter but still clinically relevant manifestations. These aggregated views emphasize the dataset’s capacity to capture both the diversity and the relative frequency of FoG events. Table 9 summarizes the number and duration statistics of FoG episodes, stratified by manifestation (shuffling, trembling, and akinesia). It is worth noting that the sum of the different events is larger than 101 (i.e., total number of FoG episodes labelled). This is due to the presence of different manifestations in adjacent parts of six FoG episodes. These events are characterized by transitions from shuffling to akinesia (1 event); from shuffling to trembling (1); from akinesia to shuffling (1); from trembling to akinesia to trembling (1); and from trembling to shuffling to trembling (2).

**Table 9:** Descriptive statistics of FoG episodes based on their manifestation.

Type	Events	Mean (seconds)	Median (seconds)	Min (seconds)	Max (seconds)	95^*th*^ perc. (seconds)
**Shuffling**	33	3.02	2.40	0.6	9.9	7.04
**Trembling**	40	5.82	4.90	0.2	24.1	12.40
**Akinesia**	37	19.98	12.40	1.8	108.68	71.65

When integrating this dataset with other PD cohorts, we recommend normalizing sampling frequencies and time axes, as well as verifying annotation formats for consistency, as done in a previous cross-dataset study^14^. In addition, it is worth noting that data were recorded from patients in the “Off” condition, to control for motor fluctuations and increase the probability of FoG manifestation. The standardized CSV structure has been designed to facilitate such integration and ensure compatibility across studies. There are no restrictions on the use of this dataset beyond the accompanying license. All data have been fully anonymized, and no personally identifiable information is included. Nevertheless, users are encouraged to follow best practices for secure data handling, particularly when combining this dataset with other clinical resources.

## Technical Validation

### Missing data

Due to empirical synchronization or transmission errors between the data collection smartphone and the IMU sensors, some data frames/packages were lost. The percentage of missing values is detailed in Table 10, categorized by sensor location and sensor type. The unavailable data ranges from 3.1% for the left ankle to the 8.2% for the gyroscope on the lower back. Missing samples in the dataset arise exclusively from BLE transmission drops between the IMU nodes and the smartphone. These drops are mainly due to limitations of the smartphone’s BLE antenna and connection capacity: as the number of simultaneously connected nodes and the sampling frequency increase, the probability of transmission loss also increases. Most missing samples occurred in short, isolated bursts (typically  < 100 ms) and showed no systematic association with specific activities, FoG events, or sensor locations, indicating that the missingness mechanism is consistent with missing completely at random. In rare cases, longer gaps—on the order of several seconds or, exceptionally, affecting an entire session for a single node—were observed, but these events were confined to one or at most two IMUs, while the remaining sensors recorded continuously. All missing values (both short and long periods) were retained in the released dataset to give users full flexibility in preprocessing. All streams underwent automated integrity checks, and timestamps allow users to interpolate or selectively exclude affected segments as needed.

**Table 10:** Rate of missing values for each sensor.

Sensor location	Sensor type	Missing values (%)
ankleR	acc	3.6
	gyro	3.6
ankleL	acc	3.1
	gyro	3.1
back	acc	7.7
	gyro	8.2
wrist	acc	3.1
	gyro	3.5

Considering sensor combinations, data from the IMUs on both ankles is available for 94.3% of samples; ankles and back in 88.7%; ankles and wrist in 89.4%; back and wrist in 87.5%; all four accelerometers in 87.0%; and all four IMUs in 84.7% of samples. Missing data are marked as *NaN* in the dataset. No interpolation method was used in the shared data.

### Freezing of gait recognition

In a previous study^31^, we examined the provided dataset to assess the performance of a random forest algorithm and a one-dimensional (1D) CNN for detecting FoG. The models were trained on 11 subjects, validated on 5 subjects, and tested on the remaining 6 subjects. The results indicated that the DL approach significantly outperformed the ML model (AUC 0.912 ± 0.019 vs 0.613 ± 0.014). Among various IMUs, those placed at the ankles yielded the best results (AUC 0.911–0.924), followed by the IMU on the lower back (AUC 0.774 ± 0.065) and wrist (AUC 0.638 ± 0.007). The gyroscope on the ankle surpassed the accelerometer on the same location (AUC 0.920 ± 0.008 vs 0.889 ± 0.013). Walking was easily distinguished from FoG (AUC 0.986 ± 0.003), whereas signals patterns during turning were more challenging to differentiate from FoG (AUC 0.851 ± 0.008). The 1D-CNN, trained on the present dataset, was further tested on two external databases^25,27^, comprising 12 and 7 subjects, respectively. Data from these datasets were resampled to 60Hz, and axes orientation and unit of measurement were adjusted to match the system configuration of the training data. Data underwent the same pre-processing of the training dataset, including band-pass filtering between 0.5 Hz and 20 Hz, and segmentation using 2s-long windows with 75% overlap (0.5s slide). When tested on the external datasets, the algorithm correctly detected 88–95% of FoG episodes.

The validity and utility of the FoG-STAR dataset were further reinforced by a recent study^16^, which investigated fine-tuning strategies for improving the performance of DL models in FoG detection. Acknowledging the inherent inter-subject variability in PD, the authors trained convolutional and recurrent neural networks under a subject-independent framework and subsequently fine-tuned them using small amounts of data from each target participant. This approach led to substantial performance gains – up to +20.9% in F1-score, +17.4% in recall, and +14.4% in precision – demonstrating that fine-tuning can effectively adapt general models to individual motor patterns. Beyond its methodological contribution, FoG-STAR has several features that can support translational research. Its multi-sensor configuration, multi-task structure, and detailed annotation of both activities and FoG manifestations provide the type of granular information needed for developing and benchmarking AI algorithms for wearable-based FoG monitoring. These characteristics also make the dataset suitable for initial work on clinical decision-support tools that aim to quantify symptom burden or monitor therapy response. Furthermore, the coverage of diverse gait patterns and postural transitions offers a basis for exploring home-based rehabilitation strategies that rely on continuous monitoring and feedback. While further validation is required before these applications can be deployed in practice, FoG-STAR provides a coherent and well-documented foundation for advancing research toward more clinically oriented and real-world solutions.

### Methodological considerations and limitations

The relatively small sample size (22 participants), the limited age distribution, and the medication state (“Off” condition) may constrain the generalizability of the findings. The modest cohort size restricts the diversity of motor profiles represented, while the age and medication state homogeneity may limit the dataset’s ability to capture the variability in FoG manifestations and movement dynamics observed across broader PD populations and conditions^40^. As a result, models trained on this dataset may require additional validation on larger and more heterogeneous cohorts to ensure robust generalization.

The distribution of FoG manifestations in our dataset differs from that reported in previous studies. In our cohort, trembling was the most frequent manifestation (40 episodes, 36%), followed by akinesia (37 episodes, 33%) and shuffling (33 episodes, 30%). These proportions contrast with Yu *et al*.^19^, who observed 15% akinesia, 41% shuffling, and 44% trembling, and with Yang *et al*.^18^, where trembling represented 83% of all episodes and akinesia 17%. Several factors may explain these discrepancies. First, the population enrolled in our study exhibited more advanced disease. Compared with Schaafsma *et al*.^35^, participants enrolled in the present study were older (mean age 70 vs. 63 years) and had substantially greater motor impairment (UPDRS-III ON score 39 vs. 10), conditions known to increase the prevalence of akinetic FoG. Second, recent ML-focused studies^18,19^ provided limited clinical characterization, either not reporting clinical data or providing only the total UPDRS, without specific UPDRS-III score. This makes it difficult to directly compare the cohort of subjects under evaluation. Third, all assessments in this study were performed in the “Off” therapeutic state and participants were selected based on frequent daily-life FoG. These conditions are likely to promote akinetic and mixed FoG manifestation. Finally, unlike previous studies that typically assign a single dominant manifestation to each episode, our annotation protocol did not impose a dominant label. When multiple patterns occurred within the same episode, all observed manifestations were retained. This resulted in some mixed patterns (e.g., trembling-shuffling or shuffling-akinesia), and this finer-grained labeling approach can slightly shift the apparent distribution of FoG subtypes. Together, these differences in disease severity, medication state, cohort characterization, and annotation granularity can contribute to the distinct manifestation distribution observed in our study.

The FoG-STAR dataset and the corresponding annotations use the term severity to describe three observable patterns within a freezing episode: shuffling, trembling in place, and complete akinesia. In the classical taxonomy^35^, these patterns are referred to as manifestations of FoG rather than clinical severity categories. In our study, we adopted the same phenomenological distinctions, but we used the term severity because the three manifestations reflect an ordinal progression in the degree of movement arrest (shuffling to trembling to complete akinesia) and therefore function as an episode-level severity proxy. This definition differs from patient-level clinical severity scored by the MDS-UPDRS, which is based on frequency and context rather than single-episode expression.

An important aspect to consider concerns the temporal resolution of the video annotations. Videos were downsampled to 10 fps, which does not allow capturing ultra-brief FoG events reported in the literature, including episodes as short as 180 ms^41^ or even 50 ms^9^. However, such extremely brief manifestations are rarely identifiable through visual inspection and, in our experience, are not consistently recognizable by clinicians during video-based FoG assessment. As clinical recognition served as the gold standard in this study, events below the threshold of the human observer would not have been reliably labeled even at higher frame rates. Moreover, the clinical relevance of sub-100 ms interruptions in gait remains uncertain, and their detection by automatic algorithms may lead to substantial false positives. For these reasons, we focused on clinically meaningful episodes and used 10 fps as a pragmatic compromise. We acknowledge, however, that this choice introduces uncertainty in the precise onset and offset timing of FoG, particularly when compared with the higher sampling frequency of the wearable IMUs.

Not all participants completed all the standardized tasks. Patients were allowed to discontinue tasks at any time. Non-completion was more likely because assessments were performed in the “Off” medication state and participants had severe motor impairment. Consequently, the number of participants performing each task varied, particularly for more demanding motor or dual-task conditions. For example, Task 5 (walking 10 meters while carrying a glass of water) was completed by only a few subjects due to the combined effect of task difficulty and patients’ motor limitations. Tasks 1, 4, and 7 (i.e., Timed Up & Go, passage through a doorway, and 360-degree turn) were the most frequently performed, with 19, 22, and 21 participants completing them, respectively. In contrast, Tasks 3 (walking) and 6 (walking while counting backward) were completed by only half of the participants (13 each). Finally, Tasks 2 (stance) and 5 (walking while carrying a glass of water) were completed by only 5 and 4 participants, respectively.

## Usage Notes

The dataset is provided in a structured format (see Section Data Records) to facilitate reuse by researchers. All files are distributed in standard formats (CSV for *sensor data* and *clinical data*), which can be easily accessed using common programming languages such as Python. To support reproducibility and accelerate adoption, we provide a set of basic scripts that enable users to: (a) Load and visualize raw sensor signals from each IMU node; (b) Inspect and plot annotated FoG episodes aligned with the sensor recordings; (c) Compute descriptive statistics (e.g., step counts, signal ranges, or task durations). These scripts are available in the repository alongside the dataset and can be executed in an environment using Python 3.12.12 together with the libraries Pandas 2.2.2, NumPy 2.0.2, and Matplotlib 3.10.0. Researchers are encouraged to adapt and extend them for advanced analyses, including gait feature extraction, FoG detection, and ML model development.

## Data Availability

The dataset generated and analyzed during the current study has been deposited in the Zenodo repository and is publicly available at https://zenodo.org/records/17838806. The dataset adheres to the FAIR principles. It includes DOI and detailed metadata (Findable); it is openly available under the Creative Commons Attribution 4.0 International license (Accessible); it includes standardized file formats (csv, python) and clear documentation (Interoperable); it comprises comprehensive description of sensors, protocols, and annotations (Reusable). All data collection procedures complied with the General Data Protection Regulation (GDPR) and received approval from the appropriate institutional ethics committee. Prior to publication, all personal identifiers were removed or pseudonymized, and only non-identifiable sensor recordings and demographic information necessary for research purposes were included.

## Code availability

To support reuse and reproducibility, we provide example scripts together with the dataset repository. These scripts allow users to load the raw sensor data, visualize annotations, and compute basic descriptive statistics (see Usage Notes for examples). In addition, the convolutional neural network (CNN) model for automatic freezing of gait recognition, developed and validated using FoG-STAR, is openly available at: https://github.com/Lu1g1n0/FoG-detection-using-a-single-ankle-mounted-sensor. Researchers are encouraged to adapt and extend both the dataset utilities and the CNN codebase for benchmarking, feature extraction, and further methodological developments. All example scripts were tested on Linux, Windows, and macOS using conda environments and Microsoft Visual Studio (Windows and macOS), as well as PyCharm and Jupyter Notebook. The scripts executed consistently across platforms, confirming cross-platform compatibility.
